# Relationship between Psychosocial Impairment, Food Choice Motives, and Orthorexic Behaviors among Polish Adults

**DOI:** 10.3390/nu12051218

**Published:** 2020-04-26

**Authors:** Marta Plichta, Marzena Jezewska-Zychowicz, Aleksandra Małachowska

**Affiliations:** Department of Food Market and Consumer Research, Institute of Human Nutrition Sciences, Warsaw University of Life Sciences (SGGW-WULS), Nowoursynowska 159C, 02-776 Warsaw, Poland; marzena_jezewska_zychowicz@sggw.pl (M.J.-Z.); aleksandra_malachowska@sggw.pl (A.M.)

**Keywords:** orthorexic behaviors, psychosocial impairment, food choice motives, adults

## Abstract

Orthorexic behaviors correlate not only with health motives when choosing food but may also coexist with psychosocial impairment. The aim of this study was to assess the motives of food choice and psychosocial impairment among adults with orthorexic behaviors through the use of ORTO-15 and ORTO-7. The data for the study were collected from a sample of 1007 Polish adults through a cross-sectional quantitative survey conducted in 2019. The respondents were asked to complete the ORTO-15 questionnaire, the Food Choice Questionnaire (FCQ), and the Clinical Impairment Assessment (CIA). Orthorexic behaviors were measured using both the 15-item and the shorter 7-item version of the ORTO questionnaire. To determine the factors coexisting with the orthorexic behaviors, linear regression models were developed. The scores of both ORTO-15 and ORTO-7 correlated positively with the global CIA scores and the scores of personal, cognitive, and social impairments, but compared to the ORTO-7 scores, the ORTO-15 scores showed weaker correlations with the global CIA score and individual CIA scales. Orthorexic behaviors measured with ORTO-15 correlated positively with such food choice motives as health, natural content, and weight control; whereas orthorexic behaviors measured with ORTO-7 showed positive bivariate correlations only with two food choice motives: health and weight control. In regression models, sensory appeal, age, and education lower than secondary were associated inversely with orthorexic behaviors measured by both the ORTO-15 and the ORTO-7. In conclusion, the obtained results confirm that orthorexic behaviors are associated with a higher score regarding health motivation and cause an increase in psychosocial impairment. In addition, orthorexic behaviors are associated with greater importance of body weight control, which confirms the relationship between orthorexic behaviors and other eating disorders (ED), such as anorexia nervosa (AN) and bulimia nervosa (BN). However, similar motives for food choice displayed by the groups with higher scores of the ORTO-15 and the ORTO-7 and strong correlation between results obtained from both tools confirmed the similarity between these two questionnaires, thus revealing the weak psychometric properties also of the shorter seven-item version of the ORTO. Future studies on food motives, psychosocial impairment, and orthorexic behaviors should consider using other tools for measuring orthorexic behaviors.

## 1. Introduction

Orthorexia nervosa (ON) is characterized by an extreme obsession with healthy eating [1]. People with this disturbed eating behavior focus on eating pure and healthy food to positively influence their health and prevent illnesses [2]. Individuals with ON often follow inflexible eating rules and show recurrent and persistent concerns related to eating as well as restrictive eating behaviors [3]. Moreover, these people practice partial fasts, as they regard them as purifying or detoxifying [3]. Their eating restrictions increase over time, until reaching a point when entire food groups are eliminated from the diet [4].

The extreme obsession with healthy eating may result in clinically significant impairment of physical, psychological, and social lives [5]. Disturbed eating behaviors are associated with social isolation, dietary restrictions, and malnutrition [4]. However, there is no apparent association with body mass index (BMI) [6,7]. Some authors suggested that a thin silhouette does not seem to be the primary motive of people with ON [4,8]. In contrast, other studies indicate that the drive for thinness is positively associated with greater ON [7,9]. Violation of self-imposed eating rules leads to excessive fear of illness, a sense of personal impurity, guilt, and shame in such individuals [10]. In addition, people with ON are at an increased risk of impairment of social or occupational functioning, as they have hardship eating with others who not believe in their eating rules [10].

Although many studies have been carried out so far with an aim of understanding the specifics of ON and the affected people, a full picture of this condition is still unavailable. On the one hand, there exists a debate about whether ON should be considered as a distinct eating disorder (ED), as a variant of an existing ED, such as anorexia nervosa (AN), bulimia nervosa (BN), and avoidant/restrictive food intake disorder, [11,12], or as an obsessive–compulsive disorder [13], or should simply be treated as a type of lifestyle [5,14].

On the other hand, a universal shared definition does not exist for ON, and no official set of diagnostic criteria has been proposed so far [15]. Moreover, psychometric instruments used in the studies, which mostly include the Bratman Orthorexia Test (BOT) [1] and ORTO-15 [16], reveal some methodological drawbacks [17,18,19]. Besides their psychometric properties, the clinical utility of these instruments is also doubtful, as the prevalence of ON reported by the indices varies between 30% and 70% [3]. Furthermore, although ORTO-15 and its derivatives (ORTO-11, ORTO-9) have been used in many studies to assess ON [3,20], it is reported [21] that these three (15-, 11-, and 9-item) versions of the questionnaire do not produce acceptable models, and therefore, should be used with caution. Thus, the next shorter version of this questionnaire, i.e., ORTO-7 was developed. Nevertheless, according to its authors, it requires further studies exploring its predictive ability against the diagnostic criteria of ON [21].

The lack of a universal definition of orthorexia encourages making an effort focused on its development and refinement. Barrada and Roncero [22] stated that orthorexia includes, in addition to the pathological dimension of ON, a non-pathological interest in healthy eating, which they called healthy orthorexia (HeOr). This approach to orthorexia was also reflected in a multidimensional measure of orthorexia—the Teruel Orthorexia Scale (TOS) [22]. The TOS measures both ON and HeOr. Whereas orthorexia nervosa has positive correlations with different measures of mental discomfort, for healthy orthorexia these correlations are null or negative, especially when controlling the effects of the pathological variant of orthorexia [22]. Key elements of healthy orthorexia are a healthy interest in diet, healthy behavior with regard to diet, and eating healthily as part of one’s identity [9]. The differences between both dimensions of orthorexia (healthy vs. pathological) should be also confirmed by different motives when choosing food. Until now, only one study has investigated the association between orthorexia and motives for choosing food [9]. Because both types of orthorexia were included in this study, it allowed for the confirmation of differences in motives predicting food choice. It turned out that in orthorexia nervosa the main motive was weight control, accompanied by sensorial appeal and affect regulation, while for healthy orthorexia the main motive was health content, accompanied by sensorial appeal and price.

We assume that orthorexic behaviors measured using ORTO 15 and ORTO-7 may exhibit both healthy orthorexia and pathological ON characteristics. That is why we expect that the higher the ORTO score, the higher will be the health motive score and the psychosocial impairment score and the lower the weight control score. Thus, the aim of our study was to estimate the occurrence of orthorexic behaviors in Polish adults using two versions of the ORTO questionnaire (ORTO-15 and ORTO-7) and their relationships with food choice motives and experiencing psychosocial impairment. To our knowledge, this study is the first to use ORTO-7 for assessing the occurrence of orthorexic behaviors. Furthermore, the use of both tools allowed determining which of them could be more useful for estimating the occurrence of orthorexic behaviors. Our study is the first in which orthorexic behaviors, motives of food choice as well as the psychosocial impairment were assessed in Polish adults.

## 2. Materials and Methods

### 2.1. Ethical Approval

This study was approved by the Ethics Committee of the Faculty of Human Nutrition and Consumer Science, Warsaw University of Life Sciences, Poland, on 29 October 2018 (resolution no. 22/2018). Informed consent was obtained from all the study participants before the study.

### 2.2. Study Design and Sample Collection

A cross-sectional quantitative survey was conducted through a research agency—ARC Market and Opinion. Participants were selected from the online consumer panel (epanel.pl) consisting of approximately 64,000 registered people. The study invitation was sent to a total of 2025 adults aged 18–65 years. Quota selection was carried out based on gender, age, place of residence, and education to ensure that the study sample represented the Polish population. During recruitment, 78 people did not complete the questionnaire during the interview, and 932 did not meet the quota requirements, while eight were removed from the database due to the absence of responses to all questions. The final study sample consisted of 1007 participants. A computer-assisted web interview (CAWI) technique was used for data collection. The average duration of one interview was 18 min. Study participants selected from epanel.pl received monetary gratification for participating in the study. To receive the remuneration, the participants had to register on the panel and complete the survey, after which they received points that could be exchanged for money. The data were collected from February to March 2019.

### 2.3. Orthorexic Behaviors

To identify orthorexic behaviors, we used the validated Polish version of the ORTO-15 questionnaire [23]. The coding method has been reversed compared to the original ORTO-15. Items 2, 5, 8, and 9 were scored as follows: 1 = “always,” 2 = “often,” 3 = “sometimes,” and 4 = “never;” items 3, 4, 6, 7, 10, 11, 12, 14, and 15 were reversely scored; while items 1 and 13 were scored as follows: 3 = “always,” 1 = “often,” 2 = “sometimes,” and 4 = “never.” The sum of scores (ORTO-15) ranged between 15 and 60 points. During data analysis, the shorter version ORTO-7 [21] was created by removing eight items (2, 5, 6, 8, 10, 12, 14, 15) from the original ORTO-15. The sum of scores (ORTO-7) ranged between 7 and 28 points. Higher scores on both scales reflected a higher occurrence of orthorexic behaviors. Cronbach’s alpha for the ORTO-15 was 0.828 and for the ORTO-7 was 0.788.

### 2.4. Psychosocial Impairment

The Polish adaptation of the Clinical Impairment Assessment (CIA), version 3.0 [24], was used to evaluate psychosocial impairment that was secondary to ED features. The CIA questionnaire had an internal consistency (Cronbach’s alpha 0.97). The items probe impairment in life domains that are typically affected by ED, which include mood, self-perception, cognitive functioning, interpersonal functioning, and work performance, over the past 28 days. CIA has three subscales to assess clinical impairment across specific domains, including personal, social, and cognitive. This tool is a 16-item self-report which is based on a four-point Likert scale (0 = “not at all,” 1 = “a little,” 2 = “quite a bit,” and 3 = “a lot”). A total CIA score is calculated to provide a global index of the severity of psychosocial impairment associated with the pathology of ED. The global CIA score was calculated by adding together the ratings on all items. The resulting score ranged from 0 to 48, with higher scores indicating greater severity of clinical impairment. Cronbach’s alpha for CIA was 0.962.

### 2.5. Food Choice Motives

The Food Choice Questionnaire (FCQ) [25] was applied to assess the motives of the food choice of participants. The original FCQ [22] contains 36 items representing characteristics namely search, experience, and credence that motivate the consumers to make general food choices. Participants rated the importance given to each item on a four-point scale (1 = “not at all important,” 2 = “little important,” 3 = “moderately important,” and 4 = “very important”). FCQ measures nine motivational dimensions, with each containing three to six items, including health, mood, convenience, sensory appeal, natural content, price, weight control, familiarity, and ethical concerns.

The FCQ was already used in over 40 countries and translated into more than 20 languages. Several studies have shown the invariance of the FCQ across cultures [26], while others present the need for adaptations of the FCQ [27,28]. Critical appraisal suggests that the original set of items should be adapted to accommodate the different cultures [26]. However, FCQ has been cross-culturally validated across several national studies, including the Polish population. Cronbach’s alphas for the different factors ranged between 0.74 and 0.90 [29]. Because in the previous studies the difficulty in identifying the 9 original factors was evident, the decision was made to use the original division of factors. However, during the analysis, the degree of matching the statements to individual scales (factors) was checked. To examine the reliability of internal consistency of the subscales we estimated Cronbach’s alpha reliability coefficient. Cronbach’s alpha equal to 0.7 has been found acceptable [30]. On this basis, a decision was made to exclude four factors from further analysis. In one factor (sensory appeal), one statement was removed to improve Cronbach’s alpha for this factor. As a result, five subscales (or factors) of modified FCQ version were taken into consideration: health, mood, sensory appeal, natural content, and weight control. Finally, scores on each scale were computed by averaging unweighted ratings for individual items. After computing, the scores were within the range of 1–4 [25]. The factors from the original FCQ and from the modified FCQ version are presented in Table 1.

### 2.6. Sociodemographic Characteristics

The questionnaire also included questions about the sociodemographic characteristics of the sample such as gender, age (in years), place of residence (village, town with 100,000 inhabitants or less, city with more than 100,000 inhabitants), and education level (primary or vocational, secondary, higher). For calculating the body mass index (BMI), the participants were asked to self-report on their body weight and height and the index was calculated as described by the World Health Organization [31].

### 2.7. Statistical Analysis

Categorical variables were presented as a sample percentage (%), while continuous ones were expressed as the mean and standard deviation. For each subscale of CIA, the obtained scores were summarized as follows: personal impairment, ranging from 0 to 18; cognitive impairment, from 0 to 15; and social impairment, from 0 to 15. The association between the total ORTO-15 score and ORTO-7 score, psychosocial impairment, and motives of food choice was assessed using Pearson’s correlation coefficient.

The associations between scores of ORTO-15 and ORTO-7 and personal, cognitive, and social impairments and motives of food choice were verified using a multiple linear regression analysis, adjusted for gender (categorical, female/male), age (continuous, in years), place of residence (categorical, village/town ≤100,000 inhabitants/city >100,000 inhabitants), education level (categorical, lower than secondary/secondary/higher), and BMI (continuous, kg/m^2^). ORTO scores treated as continuous variables were introduced into the models as a dependent variable. The independent variables included in the models were personal, cognitive, and social impairments, and motives of food choice (health, mood, sensory appeal, natural content, and weight control). Two models were created as follows: Model 1—adjusted for the set of confounders for ORTO-15 score; and Model 2—adjusted for the same confounders included in Model 1 and orthorexic behaviors measured with ORTO-7. Automatic selection of variables with the forward selection method was applied to the models. Standardized regression coefficient (*β*) and unstandardized regression coefficient (*B*) with 95% confidence interval (CI) were used. Significance was set at *p* < 0.05 for all analyses. Analysis conducted on the Model 1 and Model 2 explained, respectively, 38% and 37% of variance (*R*^2^ = 0.38; *R*^2^ = 0.37, respectively). All analyses were performed with Statistica software (version 13.3 PL; StatSoft Inc., Tulsa, OK, USA; StatSoft, Krakow, Poland).

## 3. Results

### 3.1. Sample Characteristics

The study sample consisted of 1007 participants, which included 508 female and 499 male, aged 18–65 years. Table 2 describes the sociodemographic characteristics of the participants.

### 3.2. Orthorexic Behaviors, Psychosocial Impairment, and Food Choice Motives in the Study Sample

The mean score of ORTO-15 was 37.2 ± 5.0 (standard deviation), while the mean score of ORTO-7 was 15.6 ± 3.2. The mean CIA scores determined for the three subscales were as follows: 4.3 ± 4.4 for personal impairment; 2.9 ± 3.4 for social impairment; and 3.3 ± 3.3 for cognitive impairment. The global CIA score was equal to 10.5 ± 10.6. The mean score calculated for sensory appeal as food motive was 3.0, for natural content—3.0, for health—2.9, for mood—2.8, and for weight control—2.5. The characteristics of the study sample including orthorexic behaviors (ORTO score), psychosocial impairment, and the motives of food choice are showed in Table 3.

### 3.3. Association between Orthorexic Behaviors, Psychosocial Impairment, and Food Choice Motives

A positive correlation (r = 0.854) was observed between the ORTO-15 score and the ORTO-7 score. The correlations between the scores of ORTO-15 and ORTO-7, psychosocial impairment, and motives of food choice are shown in Table 4. Both the scores of ORTO correlated positively with the global CIA scores and the individual CIA scales. Nevertheless, the ORTO-15 scores compared to the ORTO-7 scores, showed weaker correlations with the global CIA score and the scores calculated for personal impairment, social impairment, and cognitive impairment. Both the scores of ORTO-15 and ORTO-7 correlated positively with food choice motive scores. Wherein, compared to the ORTO-15 scores, the ORTO-7 scores showed weaker correlations with such scores of food choice motives as health, sensory appeal, natural content, and weight control.

The results of multiple linear regression analysis that examined the relationship between orthorexic behaviors (ORTO-15) as a dependent variable, psychosocial impairment, and food choice motives are shown in Table 5. Orthorexic behaviors showed positive associations with global CIA score and such food choice motives as health, natural content and weight control. Sensory appeal was inversely associated with orthorexic behaviors. Inverse associations were also displayed between orthorexic behaviors and age, and education lower than secondary (Table 5).

Orthorexic behaviors measured using ORTO-7 showed positive associations with global CIA score and also with health and weight control as food choice motives; while sensory appeal was inversely associated with orthorexic behaviors. Age and education lower than secondary were also associated inversely with orthorexic behaviors (Table 6).

## 4. Discussion

In this study, we evaluated a representative sample of Polish adults for the occurrence of orthorexic behaviors using the ORTO-15 and ORTO-7 questionnaires. Due to the great criticism contained in the literature about the original ORTO-15 and doubts related to the interpretation of the results obtained using this tool [6,17,18,19,22], we did not include in our study the cutoff points that were recommended by the authors of both versions the ORTO questionnaires [16,21]. The use of cutoff points in previous studies indicated that ON characterizes 30%–70% [3], and even above 80% of the studied groups [32,33,34]. These results contradict the relatively rare occurrence of ED in the general population, where prevalence rates, e.g., of AN and BN, are estimated at no more than about 2% [35]. These discrepancies result from the fact that there are no statements in the ORTO-15 that inform about disruptions in daily functioning, interpersonal stress, or health problems resulting from the use of diet [3]. The shorter version of the questionnaire ORTO-7 [21], which we used in the study, is based on the original ORTO-15 [16] and, therefore, is characterized by identical deficiencies. As a result, the assessment of orthorexic behaviors in the study group is only possible in relation to the range of behaviors included in these tools.

In our study, we observed a positive strong correlation between ORTO-15 and ORTO-7, which reveals that these two tools examine the same construct. This also means that ORTO-7 as a tool for measuring orthorexia presents the same disadvantages as ORTO-15 [6,17,18,19,22]. When using both of these tools, similar results could be expected regarding the relationship between orthorexic behaviors and food choice motives, and CIA. Indeed, both indicators correlated positively with the global CIA score as well as the scores of personal, cognitive, and social impairments. Nevertheless comparing both indicators, the ORTO-7 scores showed stronger correlations with the global CIA score and the individual CIA scales. It is worth emphasizing that the CIA is a tool that measures the level of overall depth of secondary psychosocial impairment resulting from ED [24]. Till now, the CIA questionnaire is considered as a reliable tool to assess the impact of ED symptoms on functioning in important areas of life [24,36,37]. Moreover, many authors have confirmed that ED is a significant determinant of psychosocial impairment [24,37,38]. In the study that indicated no correlation between ON symptoms and the global CIA score [39], the CIA questionnaire was modified to assess the impairment solely on the basis of eating habits. However, in the original version of CIA, exercises and nutritional considerations, figure, and body weight are included as factors affecting daily functioning, in addition to eating habits [40]. Some authors suggest that ED symptoms are the most important predictors of ON [41] and that ON may be displayed as a strategy for dealing with other EDs [11,42]. This approach can be taken into account when explaining the positive correlation between global CIA score and orthorexic behaviors of the participants. However, the correlation is weak and moderate (ORTO-15 and ORTO-7, respectively), and so the results should be interpreted with caution.

The results of our study pointed to positive correlation between orthorexic behaviors and the importance given to the motives of food choice such as health, natural content, and weight control when the ORTO-15 was used. However, when the ORTO-7 is used, the only correlations that exist are those with health motive and weight control when selecting food. The strength of the relationship between the ORTO-7 and natural content was weak. Moreover, this motive did not appear in the model created with multiple linear regression, therefore this relationship should be interpreted with caution.

The higher score of ORTO questionnaires correlated positively not only with health but also with weight control when selecting food. However, the previous studies have found that the nutritional limitations of people with orthorexic behaviors do not actually result from their anxiety about obesity, desire for weight loss, or a distorted image of the body [4,8], which are characteristics for those suffering from AN and BN [3]. Until now no correlation was found between the ON and BMI, which was the main factor used to differentiate the orthorexic behaviors from AN [43,44,45]. Nevertheless, distinguishing orthorexic behaviors from AN or BN may be more difficult in practice than in theory [39]. What is more, this belief may change in the future, as the latest studies showed that the motive for choosing food, which is weight control, was positively correlated with ON [9]. In our opinion, the greater importance given to weight control by people with orthorexic behaviors did not necessarily result from a disturbed body image. According to the current beliefs, a fit and slim body implies health, so people exhibiting orthorexic behaviors may feel the obligation to take care of their body in order to maintain correct body weight [46]. On the other hand, research indicates that consumers perceive calorie-reduced food as relatively “healthier” and tend to underestimate the calorie content of products considered as “healthy” [47]. Thus, further cultural studies focusing on perception of health and weight ideals could help to understand the association that links them with nutritional behaviors and orthorexic behaviors as well.

Additionally, both the scores of ORTO-15 and ORTO-7 correlated positively with the mood score. Researchers suggest that food consumption is generally associated with the affective state [48].

People who are in a positive mood prefer eating “healthy” food to comfort food, such as sweets or products rich in carbohydrates and fats and, thus, gain long-term health benefits that promote their well-being. On the other hand, people who are in negative moods prefer eating comfort food over “healthy” food as they may need immediate and hedonistic mood benefits [48]. However, insufficient knowledge of the cause of the orthorexic behaviors [18] and the fact that this motive did not occur in both models created with multiple linear regression makes the estimation of its impact on mood, and consequently on dietary behavior, more difficult.

It is also worth adding that in the developed models it was observed that the increase in the importance of sensory appeal caused a decrease in the severity of orthorexic behaviors (both ORTO-15 and ORTO-7). Another study also observed a negative correlation between this food choice motive and ON [9]. This is compatible to the characteristics of the orthorexic behaviors described by other studies [14,49,50]. A study by Depa et al. [9] indicated that both in the pathological and healthy dimensions of orthorexia, food’s good appearance was not a factor in its choice. Moreover for people who display orthorexic behaviors, the appearance of food may be less important than its composition and impact on health [9], which was confirmed by our results.

In our study, the intensity of orthorexic behaviors decreased with age. However, in literature, there are no unequivocal results regarding the relationship between age and these disturbed eating behaviors [50]. Until now, the study results provided information both on the absence of a relationship between orthorexic behaviors and age [43,44,51,52] and on the heightening of its intensity with age [16]. In contrast, other studies have found that younger age may determine the severity of orthorexic behaviors [53,54], which corresponds to our results. Probably this is due to a decrease in the age of people exhibiting orthorexic behaviors. Nevertheless, due to the discrepancy of results, one should refrain from drawing unequivocal conclusions.

Furthermore, inverse association was also displayed between orthorexic behaviors and education lower than secondary. Moreover, this relationship has been described in a contradicting manner in literature [50]. In some studies orthorexic behaviors were independent of education [43]. However in other studies, more severe orthorexic behaviors were found in less educated people [16,55], which was confirmed in our study, but also in people with higher education [41]. The occurrence of orthorexic behaviors in people with higher education may be explained by the fact that these people have greater nutritional knowledge and awareness of the impact of diet on their health [56,57]. Therefore these people may be more susceptible to various diets, which may result in orthorexic behaviors.

### Strengths and Limitation

Strength of this study is the use of a large sample that was nationally representative in terms of gender, age, place of residence, and education level, and therefore, the results can be generalized to the Polish population. In addition, the number of females and males in the study was similar, which allowed a reliable comparison of the results of our study by gender. Further, the lack of social control and a sense of relative anonymity on the Internet indicate that online questionnaires can be used for addressing topics that are generally considered to be embarrassing, intimate, or sensitive [58]. Therefore, in our study, we adopted the CAWI technique, which provided the respondents with a sense of anonymity and allowed them to answer freely.

Despite its valuable contribution, the study has some limitations. The data used for analysis were collected from Polish adults, and so they could not be generalized to the population of other cultural background. However, the findings could be of potential use in determining the associations between orthorexic eating behavior, psychosocial impairment, and the food choice motives of consumers in some countries that are similar to Poland. Moreover, the cross-sectional design of our study and collection of data at a single point in time did not permit conclusions to be drawn about causality. In the future, longitudinal studies should be conducted to explore how psychosocial impairment and motives of food choice vary among people with orthorexic behaviors over time. Further, multiple linear regression models, although significant (both *p* < 0.0001), accounted for only 38% (Model 1), and 37% (Model 2) of the variance of the dependent variable. Another limitation of the study relates to the potential biases that may occur when self-reported data are analyzed. The self-reported weight and height could have led to bias when BMI was calculated. In addition, the ability to self-report weight and height could be affected by sociodemographic characteristics, including age, gender, and economic status [59].

Finally, the original ORTO-15 has been questioned by other researchers [17,18,19,22], who report several inconsistencies about the validity and internal reliability of this tool. The psychometric properties of the ORTO-15 are not adequate in view of instability in its internal structure [19], low internal consistency [17,19,32], scoring scheme of the tool [19], unease about the scores interpretation [6,32,34], and problems in content validity [19]. Moreover, confirmatory factor analysis was used in the shorter version of ORTO (ORTO-7) [21]. Some researchers suggest that this technique is not appropriate, and could be considered a doubtful choice, given that the internal structure of ORTO-15 is fairly unclear [6,17]. With all of this in mind, greater attention is needed on other tools of orthorexic eating behavior, e.g., the Eating Habits Questionnaire (EHQ) [60], the Teruel Orthorexia Scale (TOS) [22], the Düsseldorfer Orthorexia Scale (DOS) [61], or on the development of new tools.

## 5. Conclusions

Our results indicate that the relationships between the assessed food selection motives, with the exception of natural content, and orthorexic behaviors measured using the ORTO-15 and the ORTO-7 were similar. In addition orthorexic behaviors measured using both of these tools positively correlated with the global CIA score. Thus, the obtained results confirm the assumption that orthorexic behaviors are associated with a higher score regarding health motivation and cause an increase in psychosocial impairment. Health motivation primarily concerns healthy orthorexia, while psychosocial impairment concerns orthorexia nervosa. Thus, we confirmed our assumption that orthorexic behaviors can indicate both healthy orthorexia and pathological features of ON. Nevertheless, we expected that orthorexic behaviors would be associated with lower importance of body weight control, which has not been confirmed. Thus, the obtained findings confirm the relationship between orthorexic behaviors and other EDs, such as AN and BN, and are consistent with the results obtained with the tools better rated in terms of psychometric properties. The importance of weight control, however, does not necessarily result from a distorted body image, but only from the desire to maintain healthy weight as a precondition for health. Further research on the use of weight control, its causes, as well as health perception in a group of people at risk of orthorexic behaviors is recommended.

The assessment of orthorexic behaviors using the ORTO-15 and the ORTO-7 showed a positive strong correlation between the results of both questionnaires. This means that the ORTO-7 has the same methodological flaws as the ORTO-15. Its use to assess orthorexic behaviors is therefore inadvisable. Therefore, it would be appropriate to repeat this study using another tool for the assessment of orthorexic behaviors, in order to identify the association between psychosocial impairment, food choice motives, and orthorexic behaviors.

## Figures and Tables

**Table 1:** Items in the original Food Choice Questionnaire (FCQ) version and short FCQ version and Cronbach’s alpha of each factor.

Original FCQ Version			Modified FCQ Version		
Items	Cronbach’s Alpha	Mean *; Standard Deviation	Items	Cronbach’s Alpha	Mean *; Standard Deviation
Factor 1—Health	0.762	2.9; 0.6	Factor 1—Health	0.762	2.9; 0.6
22. Contains a lot of vitamins and minerals		3.3; 0.7	22. Contains a lot of vitamins and minerals		3.3; 0.7
29. Keeps me healthy		3.3; 0.8	29. Keeps me healthy		3.3; 0.8
10. Is nutritious		2.9; 0.8	10. Is nutritious		2.9; 0.8
27. Is high in protein		2.5; 0.9	27. Is high in protein		2.5; 0.9
30. Is good for my skin/teeth/hair/nails, etc.		3.2; 0.8	30. Is good for my skin/teeth/hair/nails, etc.		3.2; 0.8
9. Is high in fiber and roughage		2.5; 0.9	9. Is high in fiber and roughage		2.5; 0.9
Factor 2—Mood	0.766	2.8; 0.6	Factor 2—Mood	0.766	2.8; 0.6
16. Helps me cope with stress		2.3; 0.9	16. Helps me cope with stress		2.3; 0.9
34. Helps me to cope with life		2.6; 0.9	34. Helps me to cope with life		2.6; 0.9
26. Helps me relax		2.8; 0.9	26. Helps me relax		2.8; 0.9
24. Keeps me awake/alert		3.2; 0.7	24. Keeps me awake/alert		3.2; 0.7
13. Cheers me up		2.6; 0.9	13. Cheers me up		2.6; 0.9
31. Makes me feel good		3.2; 0.7	31. Makes me feel good		3.2; 0.7
Factor 3—Convenience ^a^	0.186	2.9; 1.2			
1. Is easy to prepare		2.8; 0.9	Factor is not included		
15. Can be cooked very simply		2.8; 0.9			
28. Takes no time to prepare		3.0; 5.4			
35. Can be bought in shops close to where I live or work		3.0; 0.8			
11. Is easily available in shops and supermarkets		2.8; 0.8			
Factor 4—Sensory Appeal	0.699	2.9; 0.6	Factor 4—Sensory Appeal	0.740	3.0; 0.6
14. Smells nice		2.9; 0.8	14. Smells nice		2.9; 0.8
25. Looks nice ^b^		2.8; 0.8	18. Has a pleasant texture		2.8; 0.8
18. Has a pleasant texture		2.8; 0.8	4. Tastes good		2.8; 0.8
4. Tastes good		3.3; 0.7			
Factor 5—Natural Content	0.709	3.0; 0.7	Factor 5—Natural Content	0.709	3.0; 0.7
2. Contains no additives		2.9; 0.9	2. Contains no additives		2.9; 0.9
5. Contains natural ingredients		2.9; 0.9	5. Contains natural ingredients		2.9; 0.9
23. Contains no artificial ingredients		3.3; 0.8	23. Contains no artificial ingredients		3.3; 0.8
Factor 6—Price	0.564	2.9; 0.6			
6. Is not expensive		2.8; 0.8	Factor is not included		
36. Is cheap ^b^		2.8; 0.8			
12. Is good value for money		3.0; 0.8			
Factor 7—Weight Control	0.854	2.5; 0.9	Factor 7—Weight Control	0.854	2.5; 0.9
3. Is low in calories		2.4; 0.9	3. Is low in calories		2.4; 0.9
17. Helps me control my weight		2.5; 1.0	17. Helps me control my weight		2.5; 1.0
7. Is low in fat		2.5; 0.9	7. Is low in fat		2.4; 0.9
Factor 8—Familiarity ^a^	0.471	2.7; 0.6			
33. Is what I usually eat		2.7; 0.8	Factor is not included		
8. Is familiar		2.7; 0.8			
21. Is like the food I ate when I was a child		2.6; 0.9			
Factor 9—Ethical Concern ^a^	0.564	2.9; 0.5			
20. Comes from countries I approve of politically		1.9; 0.9	Factor is not included		
32. Has the country of origin clearly marked		2.8; 0.9			
19. Is packaged in an environmentally friendly way		3.9; 0.8			

FCQ—food choice questionnaire; * 4-point scale: 1 = “not at all important,” 2 = “little important,” 3 = “moderately important,” and 4 = “very important;” ^a^ factor is not included in the short FCQ version due to Cronbach’s alpha (α < 0.7); ^b^ item has been removed from original FCQ version due to increase of Cronbach’s alpha for particular factor in modified FCQ version.

**Table 2:** Sociodemographic characteristics of the study sample (*N* = 1007).

Variables	*N*	%
Gender		
Male	499	49.6
Female	508	50.4
Age in years (mean ± standard deviation)	41.4 ± 13.7	
Age in years		
18–24	120	11.9
25–34	235	23.3
35–44	233	23.1
45–54	186	18.5
55–65	233	23.1
Place of residence		
Village	372	36.9
Town ≤100.000 inhabitants	324	32.2
City >100.000 inhabitants	311	30.9
Education level		
Lower than secondary	395	39.2
Secondary	354	35.2
Higher	258	25.6
BMI in kg/m^2^ (mean ± standard deviation)	25.6 ± 4.7	

*N*—number of participants; %—sample percentage; BMI—body mass index.

**Table 3:** Characteristics of the study sample including orthorexic behaviors, psychosocial impairment, and food choice motives (*N* = 1007).

Variables	
Orthorexic behaviors (mean ± standard deviation)	
ORTO-15 score	37.2 ± 5.0
ORTO-7 score	15.6 ± 3.2
Psychosocial impairment (mean ± standard deviation)	
Global CIA score	10.5 ± 10.6
Personal impairment score	4.3 ± 4.4
Social impairment score	2.9 ± 3.4
Cognitive impairment score	3.3 ± 3.3
Food choice motives (mean ± standard deviation)	
Health	2.9 ± 0.6
Mood	2.8 ± 0.6
Sensory appeal	3.0 ± 0.6
Natural content	3.0 ± 0.7
Weight control	2.5 ± 0.9

CIA—Clinical Impairment Assessment.

**Table 4:** Pearson’s correlations between ORTO-15 and ORTO-7 scores, psychosocial impairment, and motives of food choice.

Items	ORTO-15 Score	ORTO-7 Score	Global CIA Score	PI Score	SI Score	CI Score	Health Score	Mood Score	Sensory Appeal Score	Natural Content Score	Weight Control Score
ORTO-15 score	-	0.854 **	0.287 **	0.303 **	0.245 ***	0.251 ***	0.399 **	0.279 **	0.143 ***	0.385 **	0.532 **
ORTO-7 score		-	0.449 **	0.444 **	0.409 **	0.406 **	0.303 **	0.259 ***	0.079 *	0.232 ***	0.486 **
Global CIA score			-	0.937 ***	0.943 ***	0.933 ***	0.07 *	0.213 ***	−0.043	−0.050	0.213 ***
PI score ^a^				-	0.809 ***	0.787 ***	0.076 *	0.190 ***	−0.012	−0.019	0.246 ***
SI score ^b^					-	0.886 **	0.049	0.198 ***	−0.064 *	−0.068 *	0.178 ***
CI score ^c^						-	0.063 *	0.214 ***	−0.052	−0.063 *	0.161 ***
Health score							-	0.662 **	0.371 **	0.639 **	0.536 **
Mood score								-	0.439 **	0.401 **	0.447 **
Sensory appeal score									-	0.409 **	0.331 **
Natural content score										-	0.539 **
Weight control score											-

^a^ personal impairment score; ^b^ social impairment score; ^c^ cognitive impairment score. * *p* < 0.05; ** *p* < 0.001; *** *p* < 0.0001.

**Table 5:** Multiple linear regression analysis of orthorexic behaviors (ORTO-15) in the study sample.

Parameter	Adjusted Model 1			
	*B*	*β*	95% CI	*p*-Value
Age	−0.03	−0.09	−0.14; −0.02	<0.001
Lower than secondary education (ref. higher)	−1.11	−0.18	−0.23; −0.12	<0.0001
Global CIA score	0.09	0.19	0.14; 0.25	<0.0001
Health	1.04	0.12	0.04; 0.19	0.001
Sensory appeal	−0.65	−0.08	−0.14; −0.02	0.005
Natural content	1.03	0.14	0.07; 0.22	<0.001
Weight control	2.15	0.36	0.29; 0.43	<0.0001

*B*—unstandardized regression coefficient; *β*—standardized regression coefficient; (95% CI)—95% confidence interval; (ref.)—reference group; adjusted for gender (categorical, female/male), age (continuous, in years), place of residence (categorical, village/town ≤100,000 inhabitants/city >100,000 inhabitants), education level (categorical, lower than secondary/secondary/higher), and BMI (continuous, kg/m^2^).

**Table 6:** Multiple linear regression analysis of orthorexic behaviors (ORTO-7) in the study sample.

Parameter	Adjusted Model 2			
	*B*	*β*	95% CI	*p*-Value
Age	−0.01	−0.06	−0.16; −0.01	0.015
Lower than secondary education (ref. higher)	−0.37	−0.08	−0.15; −0.03	0.001
Global CIA score	0.12	0.35	0.29; 0.40	<0.0001
Health	0.58	0.09	0.04; 0.16	0.001
Sensory appeal	−0.41	−0.08	−0.14; −0.02	0.005
Weight control	1.42	0.37	0.31; 0.44	<0.0001

*B*—unstandardized regression coefficient; *β*—standardized regression coefficient; (95% CI)—95% confidence interval; (ref.)—reference group; adjusted for gender (categorical, female/male), age (continuous, in years), place of residence (categorical, village/town ≤100,000 inhabitants/city >100,000 inhabitants), education level (categorical, lower than secondary/secondary/higher), and BMI (continuous, kg/m^2^).
